# Syndecan receptors: pericellular regulators in development and inflammatory disease

**DOI:** 10.1098/rsob.200377

**Published:** 2021-02-10

**Authors:** Sandeep Gopal, Samantha Arokiasamy, Csilla Pataki, James R. Whiteford, John R. Couchman

**Affiliations:** ^1^ Development and Stem Cells Program, Monash Biomedicine Discovery Institute and Department of Anatomy and Developmental Biology, Monash University, Melbourne, Victoria 3800, Australia; ^2^ William Harvey Research Institute, Barts and the London School of Medicine and Dentistry, Queen Mary University of London, Charterhouse Square, London EC1M 6BQ, UK; ^3^ Biotech Research and Innovation Centre, University of Copenhagen, Biocentre 1.3.16, Ole Maaløes Vej 5, 2200 Copenhagen N, Denmark

**Keywords:** proteoglycan, heparan sulfate, glycosaminoglycan, inflammation, stem cell, cell adhesion

## Abstract

The syndecans are the major family of transmembrane proteoglycans, usually bearing multiple heparan sulfate chains. They are present on virtually all nucleated cells of vertebrates and are also present in invertebrates, indicative of a long evolutionary history. Genetic models in both vertebrates and invertebrates have shown that syndecans link to the actin cytoskeleton and can fine-tune cell adhesion, migration, junction formation, polarity and differentiation. Although often associated as co-receptors with other classes of receptors (e.g. integrins, growth factor and morphogen receptors), syndecans can nonetheless signal to the cytoplasm in discrete ways. Syndecan expression levels are upregulated in development, tissue repair and an array of human diseases, which has led to the increased appreciation that they may be important in pathogenesis not only as diagnostic or prognostic agents, but also as potential targets. Here, their functions in development and inflammatory diseases are summarized, including their potential roles as conduits for viral pathogen entry into cells.

## Graphical abstract

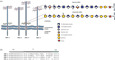

## 1. Introduction

Two small families of cell surface heparan sulfate proteoglycans (HSPGs) are present on nearly all cells of vertebrates. The transmembrane syndecans are type I membrane proteins with three or more glycosaminoglycan chains attached close to the N-terminus (i.e. distal to the cell surface [1]). By contrast, the glypicans are attached to the membrane through a glycosylphosphatidylinositol linkage and are therefore not transmembrane. They also have the potential for three or more heparan sulfate chains, but due to the globular nature of the core protein and a more C-terminal location [2], the chains are likely to be membrane proximal. The syndecans and glypicans together comprise the majority of cell surface HSPGs, though others may also be present, including a splice variant of CD44, betaglycan and neuropilin-1, though heparan sulfate (HS) chains are not always present in these cases [3]. While some experiments in invertebrates indicate partial redundancy between syndecans and glypicans [4], current evidence would suggest that this is not apparent in vertebrates. For example, of the six mammalian glypicans, deletion or mutation in three (glypicans-3, -4 and -6) give rise to developmental defects, each of which is distinct and argues for selectivity in glypican function and a lack of redundancy with syndecans [2,5].

To date, there are no known mutations in syndecan core proteins that give rise to disease in man. However, several single nucleotide polymorphisms (SNPs) in syndecan genes have been reported to associate with the disease, particularly connected to lipid metabolism. Two SNPs in SDC3 result in conservative amino acid changes in the core protein extracellular domain associate with obesity in a Korean population [6]. A non-coding region SNP in SDC4 associates with high triglyceride levels, decreased longevity, coronary artery disease and hypertension [7], while one SDC1 SNP in the 3′UTR was associated with an increased likelihood of breast cancer in an Australian caucasian female population [8]. The linkage to lipid metabolism has also been recorded in mice, where the syndecan-3 null is resistant to diet-induced obesity [9]. Moreover, the single syndecan of *Drosophila* has been shown to regulate lipid and whole-body energy metabolism [10]. Increasingly, altered expression of syndecans in a variety of diseases of the vasculature, cancers and inflammation has stimulated research into their function. It is now clear that in some well-defined cases (e.g. myeloma) mis-expressed syndecan can be a driver to disease progression [11], while in others, syndecans are strong prognostic indicators (e.g. breast cancer [12,13]). There is now much interest in syndecan functions in stem cells, which is reviewed here, along with summaries of syndecan core protein function, HS chain interactions and properties that indicate roles in development and inflammatory disease.

## 2. Syndecan distributions

In the nematode *Caenorhabditis elegans*, the single syndecan is expressed at all stages, but declines with age [4,14]. In adults, *sdn-1* mRNA expression is detected throughout the nervous system, hypodermis, germline and intestine [14,15]. SDN-1 protein expression is predominantly observed in the nervous system of adults, especially in the nerve ring [14]. Lower levels of expression are also visible in the hypodermis and vulva [14]. SDN-1 expression level is very low in adult animals, and it may not be possible to observe expression using fluorescent reporters in all tissues. Similarly in *Drosophila*, syndecan expression declines with age and has a prominently neuronal distribution, in axons, synapses and neuromuscular junctions [16–18]. A stage-specific enrichment is observed in mesoderm during gastrulation and cardiac cells during germ band retraction [19]. Proteomic analyses reveal the protein in the heart and brain [20,21].

In mammals, the distribution of syndecan-3 is reminiscent of invertebrate syndecan, with a strongly neuronal distribution [3,22]. In addition, it is present in stem cells, as discussed below and has roles in musculoskeletal development and disease [23]. Its closest relative, syndecan-1 is widespread in epithelia where it can be the dominant syndecan, for example in skin, cornea and liver [24–26]. It is also present in some lymphocyte populations, and as with the other syndecans is expressed most strongly in embryonic tissues and declines in post-natal life [3]. Experimental work has shown a linkage between syndecan-1 expression and maintenance of the epithelial phenotype, involving as yet unresolved pathways and cadherins [27].

Syndecan-4 is almost ubiquitous and is present in most nucleated cell types, though often at low levels [3]. Roles in the adhesion of mesenchymal cells to extracellular matrix have been demonstrated many times [1,28–30] and it was shown many years ago to be an early response gene sensitive to NF-kB activation and is therefore markedly upregulated in inflammatory disease [31–33]. Its closest relative is syndecan-2, typically localized in mesenchymal cells and originally known as fibroglycan [34]. Of recent interest is that some carcinomas undergoing forms of epithelial–mesenchymal transition express syndecan-2 unlike the normal parental epithelium. This has prompted research into the possibility that it may be a target in such diseases [35].

## 3. Murine syndecan knockouts and relation to disease

None of the knockouts of a murine syndecan has a lethal or severe developmental consequence, but give rise to subtle developmental defects and also impaired responses to tissue injury in post-natal life. Even a double deletion of syndecan-1 and -4 shows no severe developmental defects, suggesting that redundancy between syndecans, at least through development, may be an important property [36]. Data from studies on the epidermis show clear differentiation defects in the double knockout that are absent in corresponding single knockouts [36], supporting the hypothesis that syndecans can substitute for each other.

Table 1 lists reports from all four syndecans emanating from deletion studies, but not including cancer models. A variety of diseases have been investigated, but looking for an overall theme, many reported disease models show alterations in the vascular system and inflammation. It then appears that while vascular development is mostly unaffected through embryogenesis, the absence of a syndecan impacts tissue repair with abnormal vascular responses, probably as a result of the involvement of the immune system. The presence of a closed vascular system in vertebrates, along with the evolution of a highly complex immune system seem to have provided new roles for syndecans beyond those seen in invertebrates. This is entirely compatible with two rounds of gene duplication at the invertebrate–vertebrate boundary that has given rise to four mammalian syndecan genes [48].

**Table 1.** Syndecans in human disease and pathology. Selected examples of the use of syndecan null mice in disease models. All four syndecan deficient mouse strains develop normally, it is only when subjected to a challenge that phenotypes emerge. Cancer models are not included; references are in parentheses.

syndecan	human disease/pathology	related disease model phenotype in null mice
SDC1	inflammatory bowel disease	increased disease severity in Sdc1^−/−^ mice in dextran sodium sulfate (DSS) model of inflammatory bowel disease [37]
SDC1	bacterial infection	*Sdc1^−/−^* mice are resistant to *Pseudomonas aeruginosa* infection [38]
SDC1	ischemic injury	impaired arteriogenesis in *Sdc1^−/−^* mice in response to hindlimb ischemia [39]
SDC2	wound healing	EC specific *Sdc2^−/−^* animals exhibit impaired wound healing associated with impaired neovascularisation responses [40]
SDC3	obesity	*Sdc3^−/−^* mice are resistant to obesity when fed high fat diet [9]
SDC3	rheumatoid arthritis	*Sdc3^−/−^* mice have improved outcomes in CXCL1 and antigen-induced models of RA [41]
SDC4	inflammatory bowel disease	increased disease severity in *Sdc4^−/−^* mice in dextran sodium sulfate (DSS) model of inflammatory bowel disease [42]
SDC4	wound healing	*Sdc4^−/−^* mice have impaired dermal wound healing [43]
SDC4	osteoarthritis and rheumatoid arthritis	*Sdc4^−/−^* mice are protected in models of osteoarthritis and RA [44]
SDC4	pressure-induced heart failure	Sdc4^−/−^ mice exhibited reduced tissue repair responses in the heart following pressure overload [45]
SDC4	lung fibrosis	Sdc4^−/−^ mice protected in the bleomycin-induced lung fibrosis model [46]
SDC4	septic shock	worse outcomes are observed in mice subjected to endotoxin shock [47]

## 4. Syndecan core protein signalling

**Figure 1.**
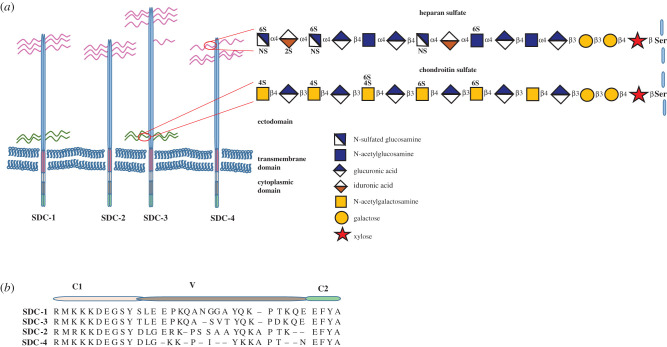
(*a*) Schematic of the overall structure of the four mammalian syndecan core proteins and the composition of their heparan and chondroitin sulfate chains. Core protein-proximal regions of the HS chains may be poorly sulfated or unsulfated and chains generally consist of highly sulfated domains interspersed with regions of low sulfation. At the interfaces between the two, there are regions of intermediate sulfation (see [33,49]). (*b*) Organization and amino acid sequences of the human syndecan cytoplasmic domains. Two regions (C1 and C2) highly conserved across all syndecans while the variable (V) regions are specific to each syndecan, yet may be highly conserved across species.

All syndecans possess a short cytoplasmic domain that is inconsistent with any intrinsic kinase or phosphatase activity (figure 1). However, it has been known for more than 20 years that syndecans can both interact with the actin cytoskeleton and signal through binding of specific proteins. Many years ago we described the cytoplasmic domains as having three identifiable regions [49]. The membrane-proximal C1 and membrane-distal C2 are highly conserved across syndecan types and species to the extent that invertebrate syndecan is recognizable by these two motifs. These highly conserved regions of syndecan cytoplasmic domains are implicated in trafficking. For example, syndecan-1 is by far the most abundant family member on the surface of hepatocytes where one of its roles is to internalize specific plasma lipoproteins that bind to the external heparan sulfate chains [25]. The C1 region, through involvement of ERK, and subsequent phosphorylation by Src and binding of cortactin, appears to promote endocytosis from membrane rafts [50]. In fact, taken as a whole, the literature indicates uptake of syndecans by almost every conceivable route, clathrin-mediated uptake [51,52] and macropinocytosis of syndecan-1 in pancreatic adenocarcinoma [53], while syndecan-2 interacts with caveolins [54]. The reasons for this variety are unresolved, but may have much to do with the accessory receptors that accompany the syndecan. The C2 domain of syndecans interacts with a number of PDZ domain proteins [55] such as syntenin. This has been implicated in the biogenesis of exosomes [20], a process also involving Src and the C1 domain [56]. Very recently, syndecan-4-syntenin-Alix complexes have been proposed as essential in targeting Escrt III to the membrane for completion of cytokinesis [57]. While this may be one mechanism, syndecan-4 null cells are competent to complete mitosis.

At first sight, major roles for syndecans, through cytoskeletal interactions in regulating adhesion and migration, seem unrelated to roles in lipoprotein uptake and other endocytic events, but there are connections. Integrin recycling, for example, involving uptake, redistribution and membrane insertion has been shown to involve syndecans [28,29], while it has long been thought that organelles such as focal adhesions resemble sites of frustrated endocytosis.

The central V (variable) regions of syndecan cytoplasmic domains have presented considerable challenges. Very little is understood regarding those of syndecans-1, -2 and -3, although many potential interacting proteins have been identified [58]. Each has a distinct amino acid sequence, while the vertebrate V regions are quite distinct from the larger V regions of invertebrate syndecans. No signalling pathway involving the invertebrate V regions has been identified to date. Best understood regarding syndecan-4 cytoplasmic domain, and we were able to demonstrate a pathway involving protein kinase C*α* [1,3,48]. The cationic V region can interact with inositol phospholipid (PtdIns4,5P_2_) that induces a conformational change, imaged by NMR spectroscopy [59], which allows binding of protein kinase C*α* in an active state. In turn, there are several potential substrates that have downstream functions in the actin cytoskeleton [60,61] and calcium regulation. One PKC-dependent substrate is the stretch-activated TRPC7 channel that associates with syndecan-4 and α-actinin [36]. Overall, it seems that syndecan-4 plays a key role in regulating the channel and when brought into play, there is a decrease in cytosolic calcium levels. Others have suggested the closely related TRPC6 can also be regulated by syndecan-4 [62]. Consistent with this, molecular and genetic analysis suggests that syndecan-1 and -4 have roles in cell adhesion, junction formation and cell migration in part through TRPC channels. It provides a distinct adjunct to integrin-based functions with which the syndecans are often associated and suggests that syndecans can be sensors of mechanical stresses. Genetic experiments in *C. elegans* suggest this property is ancient and conserved [36].

The diverse functions of syndecans are exemplified by a large number of potential extra- and intracellular binding partners, summarized recently [58]. The four syndecans have in total of 351 potential binding partners. Out of these, approximately 100 are likely to interact with HS chains [58]. The four syndecan core proteins share 18 binding proteins including themselves as they are capable of forming homo- and hetero-oligomers. They are largely cytoplasmic and include protein kinases (Fyn, Src), actin network organizers (cortactin syntenin-1, neurofibromin), *α* and *β* tubulin, the transport protein synbindin, and proteins involved in different signalling pathways (CASK, synectin, GIPC-1, TIAM1, the transmembrane integrin *α*6*β*4 and HS-binding FGF2. The 74 syndecan-1-specific binding proteins are mainly related to integrin and growth factor/cytokine signalling pathways, and interestingly, syndecan-1 is the only syndecan family member suggested to interact with fibrillar collagens I, III and V. There are also more reports of pathogens binding to syndecan-1 than other family members, but since it is also the most extensively studied member of this family, most likely our knowledge is far from complete. 25% of the 56 syndecan-2-specific binding partners are proteins associated with lysosomes while the 11 binding partners of syndecan-3 include molecules involved in cell communication and transduction, axon guidance and by interacting with the Sulf-1 and Sulf-2 sulfatases, has a regulatory role in post-export HS editing. 71% of syndecan-4-specific binding proteins are implicated in integrin signalling, the rest are extracellular or associated with exosomes with roles in cell communication [58]. Another review from 2019 uses bioinformatics tools to predict binding partners of syndecans [63].

## 5. Heparan sulfate and the ligand paradox

The structure and synthesis of HS have been well covered previously [64]. A schematic shows the overall structure of the heparan and chondroitin glycosaminoglycans (figure 1). A notable property of these polysaccharides is the presence of sulfate and uronic acid residues that impart strong anionic properties. Unsurprisingly, therefore, many proteins with clusters of basic amino acids have the potential to interact with HS chains. In some cases, a very precise fine structure of HS in terms of sulfation is required for interaction, the best example being antithrombin III [64]. For many other ligands, however, lower levels of specificity in HS fine structure apply [65,66]. In the clinical setting, heparin and its ability to bind antithrombin III is of great importance. Heparin is a specialized form of HS with high levels of sulfation including a specific 3-*O*-sulfate moiety [64,67]. It is initially synthesized as a proteoglycan, the core protein being serglycin. Subsequently, the heparin chains are cleaved to generate oligosaccharides. As well as its use as an antithrombotic agent, it is used in research as a readily obtainable model glycosaminoglycan, and there is abundant literature where particular proteins are described as having heparin-binding domains. In most cells and tissues, however, HS of lower sulfation, attached to core proteins of the syndecans, glypicans and basement membrane proteoglycans are the sites of most physiologically relevant ligand interactions [65,68]. An intriguing question, not fully answered is to what extent *in vivo* there is uniformity of, for example, skin keratinocyte syndecan-1 HS chains and how this changes through development, tissue repair and tumourigenesis. Whether fibroblast syndecan-4 HS chains are distinct from those of syndecan-2 or glypican in the same cell *in vivo* is unknown, but some HS-directed antibody studies certainly suggest that there are tissue and cell-specific HS chains [69,70]. The organization of sulfated domains of glycosaminoglycan chains *in vivo* is far from random, but the extent of variance is unclear.

Hundreds of proteins have been shown to bind heparin (and/or HS). Major families include chemokines, cytokines, extracellular matrix proteins and collagens, morphogens, growth factors, mediators of lipid metabolism and a variety of enzymes. In addition, as described below the number of pathogens, most notably viruses bind to cell surface HS. Several bacteria have been described to use syndecans for infectivity, including *Bacillus anthracis, B. cereus, Listeria monocytogenes, Pseudomonas aeruginosa* and *Streptococcus pneumonia*e [71]. In addition, much work has focused on *Plasmodium*, the malarial parasite [72].

With so many potential ligands, the question arises how protein interactions with HSPGs can generate specific information for cells and tissues? In many cases, further receptors are involved, with the frequent observation that ternary complexes of ligand, HSPG and other receptor are functional. Examples include fibroblast growth factor receptors, integrins, frizzled receptors, vascular endothelial growth factor receptors (VEGFRs) and Slit/Robo ([1,28,30,73,74] and see below). Nonetheless, syndecans are transmembrane and can signal in their own right. Therefore, it appears that ligands interacting with HS chains of syndecans impart a common set of signals, some at least influencing the actin cytoskeleton. This is consistent with data from developmental studies in invertebrates and lower vertebrates, knockout and transgenic mice, and *in vitro* experiments that point to roles for syndecans in adhesion, migration and polarity.

## 6. Heparan sulfate and fibroblast growth factors

In their review, Matsuo & Kimura-Yoshida [75] suggest that the spatio-temporal distribution, sulfation pattern and length of HS chains modulate the binding and signalling activation for different growth factors and their distribution during morphogenesis. In these processes, the cell surface HSPGs can function as co-receptors and endocytosis mediators.

One of the earliest examples of cell surface HS requirement for growth factor activity involved the fibroblast growth factor family [75]. An extensive literature has now developed over the past 30 years [76–79]. The extensively sulfated HS chains can promote the ternary complex formation with FGF (fibroblast growth factor) and its receptor FGFR (fibroblast growth factor receptor) leading to enhanced FGF signalling [80] while the desulfation of HS downregulates FGF signalling activity [81,82]. An important principle has emerged from studies of FGF2/FGFR and HS. A minimal HS pentasaccharide is required for binding FGF2 but this is not mitogenic. A longer (at least decamer) HS oligosaccharide is required that includes a 6-O-sulfated region binding to the FGFR. In this way, a ternary complex of HS/FGF2/FGFR is stabilized for signalling and mitogenesis. FGF proteins can be stably co-localized with syndecan-1 [83] while in macrophages syndecan-2, selectively binds FGF2 in a form that trans-activates receptor-bearing BaF3 lymphoma cells transfected with human FGFR [34]. In human metastatic melanoma cell lines, both CS and HS-bearing proteoglycans were shown to be partners of bFGF-mediated proliferation [84,85]. These interactions are not exclusive to cell surface proteoglycans since basement membrane perlecan also binds bFGF through its HS chains [86]. Perlecan has been also described to form ternary complex with FGF18 and FGFR3 in a HS-dependent manner during cartilage development [87].

## 7. Heparan sulfate: post-translational editing

HS can be subject to two distinct types of modification once on the cell surface. Their effects are not limited to syndecans, but can extend to any cell surface HS. The heparanase-1 endoglycosidase enzyme [88] cleaves the chains into oligosaccharides, which if large enough can retain biological activity. Released oligosaccharides therefore can serve as competitors for the binding of growth factors and cytokines, for example. Moreover, heparanase-1 can promote signalling and regulate transcriptional events, exosome formation and autophagy in promoting cell survival [89]. The expression of heparanase is regulated and known to be increased in several different types of cancer [88,90]. Heparanase inhibitors have been developed, some of which are in trials as cancer therapeutics [89,90]. Less is known about a homologue, heparanase-2, though it has no enzymatic activity, and may be a heparanase inhibitor [91].

There are also two mammalian sulfatases (Sulf1 and Sulf2) that selectively remove some 6-O-sulfates from heparan sulfate chains [92]. This can have the effect of modifying the affinity of heparan sulfate for binding ligands and again there is evidence for upregulation, notably of Sulf2, in some types of cancer [93].

## 8. Heparan sulfate and Wnt signalling

The Wnt signalling pathway is one of the most conserved pathways in metazoans, with an important role during embryogenesis as well as in maintaining tissue homeostasis in adult organisms by promoting tissue renewal and reorganization [94]. As with many morphogens and growth factors, Wnt signalling can also be subverted in disease. In a Wnt-1 model of mammary carcinoma in the mouse, syndecan-1 was shown to be essential [95]. Wnt signalling has two main branches: the canonical Wnt/β-catenin and the non-canonical pathways that can be further divided into planar cell polarity and calcium pathways. The first evidence of involvement of GAG chains in Wnt signalling came in 1997 from *Drosophila* experiments and the involvement of glypicans and syndecans in Wnt signalling has been shown in many other model organisms [94,96]. There are multiple points at which HS chains can be involved in Wnt signalling, going beyond the fact that Wnt ligands have heparin and HS-binding ability. In addition, evidence suggests that syndecans and glypicans at the cell surface can mediate Wnt signalling, but to what extent these are redundant pathways is not yet clear. In several instances, including *Xenopus* planar cell polarity processes, foregut formation and gastrulation, also muscle satellite cells, a functional complex of Fzd7 and syndecan-4 has been noted [97–100].

Desulfation of HS by Sulf1 and Sulf2 6-O-endosulfatases promotes the binding of Wnt ligands to Fzd (Frizzled) receptors [101]. Desulfation of HS on glypican-1 results in a decreased affinity of Wnt-HS interactions with indirect facilitation of Wnt-Fzd complex formation [102]. Glypican-3 directly interacts with Wnt and Fzd through GAG chains [103]. In hepatocellular carcinoma (HCC) cells and mouse models, researchers identified the Wnt binding domain on glypican-3 as being a phenylalanine 41 residue in the hydrophobic groove in the N-lobe and both the core protein and HS chains can activate Wnt-β-catenin signalling [104]. Syndecan-1's HS chains promote cell proliferation by directly binding Wnt3a and activating paracrine Wnt-Fzd signalling in multiple myeloma [105]. Moreover, Ren *et al.* [105] demonstrated that knockdown of EXT1 (critical component of the polymerases in HS synthesis) mediated aberrant Wnt/β-catenin pathway activation in melanoma. In addition, the R-spondins can bind HS. These extracellular proteins through interaction with Lgr4–6 proteins lead to the suppression of Fzd ubiquitination by the closely related ZNRF3/RNF43 E3 ligases [106]. However, recent data suggested that R-spondins may function in the absence of Lgr receptors, providing HSPGs were available [107]. In at least two ways, therefore, HSPGs can mediate and amplify Wnt signalling.

Glypican core proteins are unrelated to those of syndecans and undergo modifications that influence Wnt activity in ways not shared with syndecans. Notum was previously thought to act as a phospholipase cleaving *Drosophila* glypicans and thus regulating the distribution of Wnt [108]. However, Vincent's group in 2015 has shown that Notum requires glypicans, by virtue of interaction with HS chains, to suppress Wnt signalling, but not by cleaving their GPI anchor [109]. As revealed by kinetic and mass spectrometric analysis of human proteins, Notum acts as a carboxylesterase that removes the palmitoleate moiety of Wnts, which is important for receptor binding and in this case, therefore, glypican acts as a negative regulator of Wnt [109]. Recent work also suggests that glypican-6 may inhibit Wnt5a in gut development [110]. However, a very recent report illustrates the complexity of HSPG-Wnt interactions. A subset of glypicans (e.g. Dally-like in *Drosophila* and mammalian glypicans-4 and -6) may undergo a conformational change on binding Wnt that provides a hydrophobic pocket for the morphogen's lipid moiety. In this way, the morphogen can be dispersed and promote Wnt signalling [111].

Hedgehog (Hh) proteins are a small family of morphogens that are important for many aspects of embryonic development and are implicated in several diseases [112,113]. As with Wnts, they are lipoglycoproteins. Mammals express 3 Hh proteins: Sonic Hedgehog (Shh), Indian Hedgehog (Ihh) and Desert Hedgehog (Dhh). The literature concerning syndecan interactions with Hh is sparse, in contrast with that with glypicans. It has been shown that syndecan-4 HS can bind Shh via a cationic motif (lysines 32–38) and lysine 178 in pancreatic ductal adenocarcinoma (PDAC) and pancreatic cancer (PANC1) cells [114]. Glypican-5 binds to both Hh and its receptor Ptc1 (Patched 1) via GAG chains [115], and perlecan can also function as a Shh coreceptor [116].

Unlike HS chains, the biological function of syndecan chondroitin sulfate chains (CS) is less understood. It has been suggested that there is a cooperative role of CS and HS of syndecan-1 in laminin binding [117]. In mouse mammary epithelial cells, the CS of syndecan-1 helps the faster binding and release of FGF2-FGFR1 complex from the HS chains [118,119]. Syndecan-1's CS chains also promote Slit signalling in axon and myotube guidance [120,121]. The CS chains of syndecan-1 are membrane-proximal and this region of the core protein is also commonly a target of metalloenzymes that can shed syndecans [122,123]. A further untested possibility is that the CS chains shield this region from cleavage. It has been noted previously that clipping of HS chains by heparanase can render syndecans more susceptible to protease cleavage [124].

## 9. Syndecan shedding

The membrane-proximal core proteins of the syndecans are exquisitely sensitive to a number of proteases. *In vivo*, the most likely candidates are matrix metalloproteinases (MMPs) and ADAMs (a disintegrin and metalloproteinase). Since metalloenzymes can be upregulated under conditions of inflammation, it is not surprising that there are many reports of syndecan shedding in tissue injury. This area has been reviewed recently [123]. There can be several outcomes of these events. On one level, the shed proteoglycan, if it retains glycosaminoglycan chains, can act as a competitor of cell surface events [123]. However, in some cases, the shed proteoglycan may present bound ligands to other surface receptors, for example, with *α*4*β*1integrin and vascular endothelial growth factor receptor 2 [125]. In addition, specific regions of the external core protein of syndecans have properties independent of glycans and can inhibit syndecan-driven events [125,126]. The term ‘synstatin’ has been coined to describe such inhibitory ectodomain polypeptides, notably of syndecan-1, and these have been shown to inhibit such processes as angiogenesis [125,127].

Cleavage of the ectodomain leaves behind the syndecan transmembrane and cytoplasmic domains, the fate of which is largely unclear. There is a single report that this can be subject to y-secretase cleavage, releasing the cytoplasmic domain into the cytosol [128]. Another report suggests signalling through protein kinase C*γ* to FAK and ERK to further increase MMP synthesis [129]. However, information is sparse and in part this may be due to technical issues with tracking the fate of these small cytoplasmic domains. Syndecan-4 cytoplasmic domain has a distinct dimeric structure [59], easily disrupted by tag insertion. Tagging the C-terminus may result in preventing this C2 region from interacting with PDZ domain proteins such as syntenin or CASK [55–57,130], which could disrupt normal trafficking. However, since the cytoplasmic domains of syndecans link to the cytoskeleton and calcium channels, it would be interesting to know how shedding impacts these interactions.

## 10. Syndecans in invertebrate development

Roles for HSPGs, including syndecans, in development were originally suggested over three decades ago [131]. While syndecan knockout mouse models do not result in marked developmental defects, mutants in invertebrate models and zebrafish showed significant defects [14,132]. However, all invertebrates of the Bilateria express only a single syndecan core protein [133,134]. Therefore, its loss may be expected to have more impact on development. Zebrafish and other bony fishes do not express syndecan-1 and therefore have three core proteins, alongside the glypicans.

The core protein of invertebrate syndecans has limited sequence similarity with mammals, except in the cytoplasmic domain. The cytoplasmic domains of *C. elegans* and *Drosophila* syndecans have a high degree of sequence homology with mammalian syndecans, notably in the C1 and C2 regions. Therefore, it is possible that the signalling through the cytoplasmic domain is conserved across species, though very little is known about signalling through invertebrate cytoplasmic domains. For instance, *C. elegans* syndecan (SDN-1) cytoplasmic domain undergoes phosphorylation at a serine residue in the membrane-proximal C1 region. This is similar to the previously shown phosphorylation of Ser179 in mammalian syndecan-4 [135] that may be important for receptor recycling. SDN-1 is expressed widely in *C. elegans* including the nervous system, intestine, hypodermis and germline, and controls egg laying and the development of neurons and germlines [4,14,136,137]. The expression of *sdn-1* mRNA appears to be very high in embryos and moderate in early larval stages but low in young adults [138]. In adults, the highest expression of *sdn-1* is observed in the nerve ring, nerve cords and the vulva. Similarly, *Drosophila* syndecan (Sdc) is expressed from embryo through larval stages to adults, with the latter showing strong expression in nervous, circulatory, digestive and endocrine systems [139,140]. While the loss of *sdn-1* is not associated with lethality in *C. elegans*, Sdc mutants showed partial lethality in *Drosophila*.

**Figure 2.**
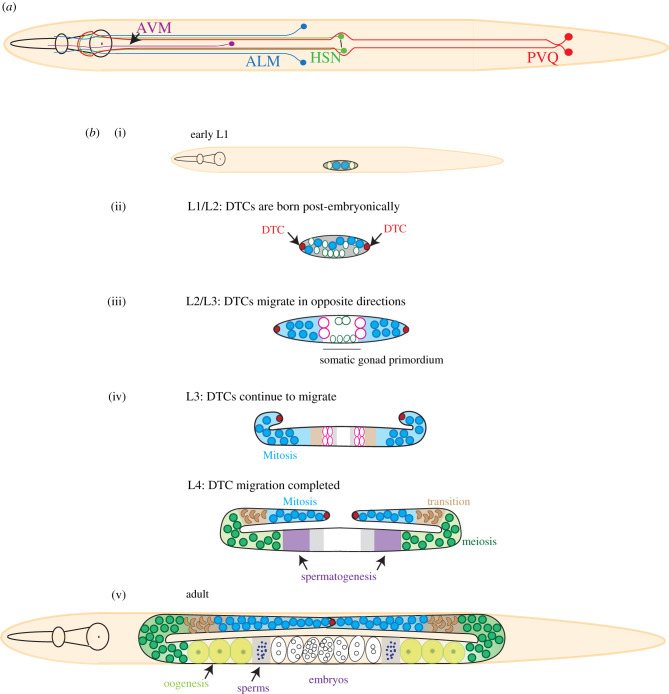
(*a*) SDN-1 controls neuronal migration during *C. elegans* development. The image shows a selection of SDN-1 regulated neurons. (*b*) Schematic showing germline development in hermaphroditic *C*. *elegans*. SDN-1 is required for the correct positioning of the distal tip cells (DTC). Two DTCs appear post embryonically and migrate in opposite directions from somatic gonad primordium until L4 larval stage. DTCs acts as a niche for germline stem cells in *C. elegans* and promote proliferation of stem cells. The hermaphrodite germline first completes the production of sperm at L4 stage before switching to oocytes in young adults. The sperm stored in spermatheca fertilizes the oocytes from which embryos develop.

Previous reports have shown that loss of the syndecan (SDN-1) in *C. elegans* has significant effects on the development and behaviour of the organism. Early studies in *C. elegans* revealed roles for SDN-1 in neuronal development. Hermaphrodite specific neuron (HSN), anterior lateral microtubule (ALM), anterior ventral microtubule (AVM), PVQ and amphid interneurons (AIY) are a few examples of SDN-1 controlled neurons during development [4,14] (figure 2). In addition to a single syndecan HSPG, *C. elegans* genome also encodes two glypicans (*lon-2* and *gpn-2*) and one perlecan (unc-52) and several CS-bearing proteins [141]. However, the HS-bearing status of UNC-52 is debated. HSPGs acts redundantly during neuronal development (e.g. AIY neurons), at least in the case of SDN-1 and LON-2. However, triple mutants for *sdn-1*, *lon-2* and *unc-52* showed significantly increased developmental defects in multiple neurons compared to single mutants or *sdn-1* and *lon-2* double mutants. This suggested UNC-52 may have functions in independent pathways [137,142]. The other glypican, GPN-1, does not appear to have any effect on neuronal development on its own. However, together with SDN-1 it appears to control ventral neuroblast migration through Kallmann syndrome protein (KAL-1) in a HS chain-dependent manner [143].

The majority, if not all of the neuronal defects resulting from loss of SDN-1 are due to an impaired migration of the cell or cell type. The migration defects can be decreased or increased migration, or guidance defects (directionality) [14]. For instance, HSN cell bodies follow specific migration pattern and axons are extended in a stereotypic manner in wild-type worms [14,36]. In the absence of syndecan, HSN cell bodies failed to migrate to their correct position and outgrowing axons were misguided [14,36]. HSN migration appears to be a Wnt signalling-dependent process, where the wnt ligand EGL-20 plays a crucial role. It has been reported that *egl-20* requires SDN-1 to mediate HSN migration. Similarly, anteriorly directed extension of PVQ neurons is often misguided in *sdn-1* mutant worms [144,145]. Consistent with data from mammalian cell systems, both HSN and PVQ defects in *sdn-1* mutant worms were shown to involve dysregulated calcium metabolism in these neurons [36]. It is also reported that loss of *lon-2* in *sdn-1* mutant can further enhance the defects in HSN development and axon branching, though any role for LON-2 in regulating calcium is unclear [146]. *Drosophila* also exhibits neuronal anatomy defects in the absence of Sdc, the proteoglycan being required for the development of CNS in embryos by regulating the Slit family of secreted extracellular matrix proteins [18,147]. Interestingly, the cytoplasmic domain of syndecan appears to be dispensable for syndecan controlled slit signalling [49]. This is consistent with the findings that both slit and its receptor, robo exhibit binding to heparan sulfate that is essential for function [18,147,148]. Moreover, it has been suggested that HS deficiency, with impact on slit/robo signalling, may be associated with the autistic phenotype in humans [149]. Finally, Sdc along with glypican appears to control neuromuscular junction development in *Drosophila* through a tyrosine phosphatase mediated process [19,150].

## 11. Syndecans and vertebrate neuronal development

Studies in vertebrate models have revealed a similar role for syndecans in neuronal development. The roles for vertebrate syndecans in the nervous system appear to be fulfilled mostly, but not exclusively, by syndecan-3. The syndecan-3 knockout mouse nervous system develops normally, except for subtle defects. For example, there was delayed radial neuronal migration in the cortex, which was rectified within ten days after birth [22]. In order to control neuronal migration in the mouse brain, syndecan-3 appears to signal with Src kinases, cortactin and EGFR25. Vertebrate success is, in part, due to the origination and plasticity of the neural crest. In zebrafish, the migration of neural crest cells is controlled [151]. Similar to the single syndecan in *C. elegans*, syndecan-4 is expressed throughout the early embryonic stages of zebrafish [152]. Syndecan-4 knockdown in zebrafish resulted in an excessive proliferation of neural cells and aberrant branching of axons. Impaired axon branching indicates defective migration of cells during development, which is therefore similar to invertebrate models [152]. These studies collectively show that both vertebrate and invertebrate syndecans are required for neuronal development. However, the loss of function mutants exhibit less severe phenotype in vertebrates, particularly mammals, perhaps due to the redundancy between syndecan isoforms.

## 12. Nematode germline development

In addition to neurons, germline development in *C. elegans* is influenced by SDN-1 [153]. *C. elegans* germline development requires the controlled migration of a special cell called the distal tip cell (DTC), which later acts as a stem cell niche for germline stem cells in adult worms. DTCs in embryos have positional specificity along with somatic gonad and migrate to the required position during larval growth (larval stage L1 to L4) to complete germline development (figure 2). During this process, signals from DTC promote the proliferation of germline cells [154]. It has been reported that RNAi targeting *sdn-1* results in defective migration of the DTC [153]. It is possible that this could have resulted in a defective germline, supported by the finding that sdn-1 mutant hermaphrodite worms showed a significant reduction in the number of offspring [121]. While glypican mutants in *C. elegans* did not have any reported germline defects, they also produced a lower number of offspring. Similarly, one mutation in *Drosophila* Sdc resulted in semi-fertile females suggesting possible germline defects [132]. Currently, no data is available on the effect of syndecan loss on the mammalian germline, but single-knockout mice are fertile and can reproduce.

## 13. Syndecans in stem cells

HS proteoglycans are expressed ubiquitously in stem cell niches and play an important role in controlling stem cell fate. While significant information about the role of syndecans during development came from invertebrate models, the role of syndecans in stem cells has been elucidated mostly in vertebrates. Similar to several other signalling pathways, syndecan-mediated signalling in stem cells can be initiated by HS chain interactions with ligands. Among the plethora of pathways controlling stem cell development, syndecans appear to regulate Wnt, BMP and Notch signalling [155,156]. It has been well established that syndecans are involved in tissue regeneration, wound healing and cancer progression [3,157]. The cells under these conditions have remarkable similarities to stem cells where they proliferate quickly and undergo morphological and transcriptional changes [30,158,159]. Therefore it is likely that syndecans control the same signalling in stem cells as they do during tissue regeneration and cancer development.

Initial reports documented the expression of syndecans in mouse bone marrow cells, suggesting a possible role for syndecans in hematopoietic stem cells [160]. However, syndecan functions in stem cells were elucidated in detail using muscle and neuronal stem cell models. The resident population of stem cells in muscles, the satellite cells, must be activated in response to injury in order to initiate muscle regeneration [161]. Syndecan-3 and syndecan-4 are expressed in the satellite stem cell niche, whereas syndecan-1 is absent in post-natal muscles [162,163]. In general, the expression of HSPGs is downregulated during satellite stem cell activation. This suggests that HSPGs are required for maintaining satellite cell quiescence [155,162]. However, syndecan-4 is an exception, where it is upregulated in active satellite cells. It appears that syndecan-3 and syndecan-4 are essential for muscle regeneration and perform distinct functions in satellite cells. Syndecan-4 knockout satellite cells failed to activate and resulted in an impaired regeneration of muscles after chemically induced muscle injury [162,164]. On the other hand, syndecan-3 null satellite cells exhibited abrupt differentiation post injury [165]. Notch signalling was identified to be the key pathway controlled by syndecan-3 during muscle regeneration [155].

In differentiated rat neuronal stem cells, syndecan-3 expression is upregulated when differentiation is induced by retinoic acid [166]. This indicated a possible role for syndecan-3 in neural stem cell differentiation. More recent data suggest that syndecan-1 is expressed highly in neural progenitor cells and knockdown of syndecan-1 results in reduced neural progenitor cell proliferation during cortical neurogenesis. Canonical Wnt signalling is a key pathway that controls cortical neurogenesis. The significant reduction in Wnt signalling in response to syndecan-1 silencing was identified as the reason for reduced neural progenitor cell proliferation [156]. Both muscle and neuronal stem cell models associated with the expression of syndecans with proliferation. Accumulated data suggest that syndecans function during stem cell development at least through BMP, Wnt and Notch, all of which bind HS. However, to elucidate the breadth of syndecan-mediated signalling in stem cells and their use as a marker for a particular stem cell population will require further study.

## 14. Syndecans in inflammation and tissue repair

### 14.1. Heparan sulfate proteoglycans and the endothelial glycocalyx

A defining feature of the endothelium is the luminal glycocalyx, which is a complex assemblage of sugars decorating the surface of the endothelium. The glycocalyx modulates vascular tone and permeability, as well as mediating inflammatory events. A major proportion of the sugar content of the glycocalyx is HS and despite the existence of several model systems in which the HS polymerase enzymes EXT1 and EXT2 are deleted, there is limited information as to the impact this has on glycocalyx structure and function. Studies would suggest that they, and by extension HS, are important for maintaining both endothelial cell homeostasis and glycocalyx repair after insult [167,168]. The production of heparanase by leucocytes has attracted much attention based on the premise that glycocalyx degradation is a necessary step for efficient leucocyte transmigration. However, neutrophil and effector lymphocytes do not require this enzyme or indeed HS for efficient traversal of the endothelium, whereas there does appear to be some requirement for this enzyme in monocyte and macrophage transmigration [169–171].

The principal HSPGs found on the luminal surface of endothelial cells (ECs) are the transmembrane syndecans-1, -2 and -4, and membrane-bound glypican-1 [172,173], in addition to several secreted HSPGs and a range of other glycoproteins. Loss of syndecan-1 results in a thinner glycocalyx [174], although the impact of the other HSPGs on this parameter has yet to be established. Numerous studies have identified shed syndecan-1 as a marker of both endothelial dysfunction and glycocalyx degradation, an example being ischaemia-reperfusion injury [175]. Blockade of syndecan-1 shedding led to a less inflammatory phenotype in a model of ulcerative colitis [176]. Although other HSPGs are also shed under these circumstances, they are not regarded as robust biomarkers for EC dysfunction.

### 14.2. Heparan sulfate proteoglycans and leucocytes

Genetic deletion of syndecan family members results in phenotypes which mostly become apparent when these animals are challenged. There are many studies in which syndecan null animals have been tested in disease models where there is a significant inflammatory component. Despite this, relatively little is known about the impact of syndecan deletion on factors such as leucocyte subset numbers, or indeed the extent of syndecan expression on different leucocyte cell types.

**Figure 3.**
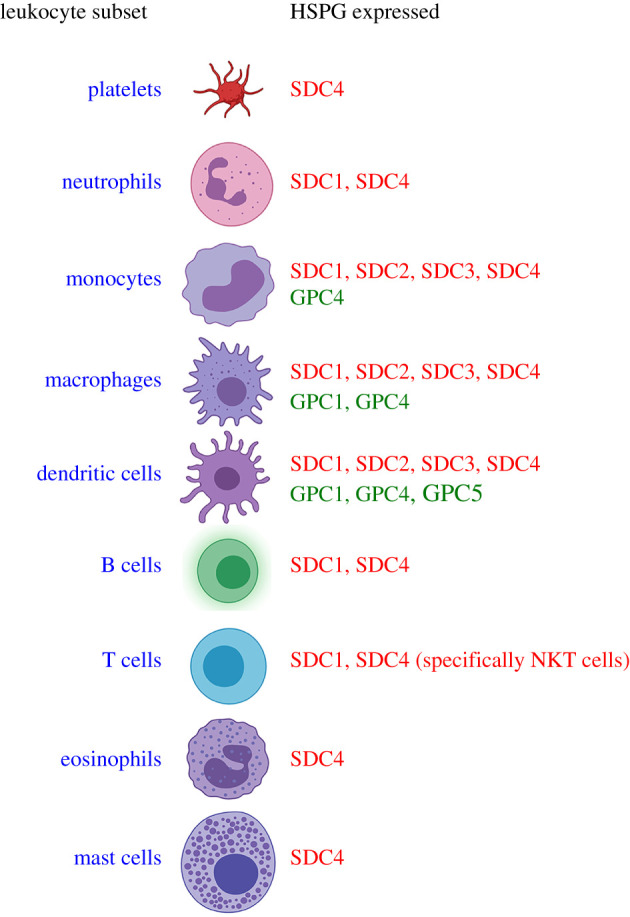
Leucocytes express a diverse range of syndecans (red) and glypicans (green). It is interesting to note that cells which are intimately associated with producing chemokines and cytokines such as macrophages and dendritic cells possess the most diverse portfolio of HSPGs. Leucocyte representations were generated with the aid of www.biorender.com. For references see [177–193].

An essential early process in wound healing is the initiation of clotting, which is primarily driven by platelets. Syndecan-4 is the only HSPG to be identified on these cells and its loss either by shedding or pharmacological blockade led to enhanced clotting responses primarily due to the fact that it bound antithrombin [177]. Neutrophils are the most abundant white blood cell and are recruited first to sites of inflammation, and there is evidence that they express both SDC1 and SDC4, albeit at low levels [156,178]. Monocytes and macrophages express all four family members, although the situation is complex [179–181]. Human monocytes can be differentiated into either macrophages or immature and mature dendritic cells. Syndecan-2, -3 and -4 mRNA can be detected at all stages of this differentiation process; however, SDC1 mRNA is only apparent in immature dendritic cells (DC). This study also reported the complete absence of glypican-6 at all differentiation stages in contrast with glypican-4 which was evident in all. Glypican 5 appears in DCs at all stages, but not monocytes or macrophages, while glypican-1 was absent only from monocytes [182]. A number of other studies confirmed the presence of all four syndecans on DCs by flow cytometry [183–185]. This expression data raises the possibility that at least in the context of monocytes, macrophages and DCs there are likely to be multiple GAG-bearing molecules on the cell surface, conceivably bearing GAG chains with distinct, core protein-specified properties. The situation is further complicated by the fact that syndecan expression is modulated by inflammatory stimuli and these same stimuli can promote syndecan ectodomain shedding. Innate lymphoid cells such as B and T cells also express syndecans. In both syndecan-1 and syndecan-4 null animals, elevated levels of NKT cells are observed [186]. In syndecan-4 null mice this is linked with the absence of N-sulfation and appears protective in a Lewis lung carcinoma model [185]. Absence of syndecan-1 correlates with an increased inflammatory response in models of psoriasis, and this is linked to the elevated levels of a subset non-SDC1 expressing γ*δ* T cells [187]. Syndecan-1 expression is associated with a number of B cell populations specifically terminally differentiated antibody-secreting cells and is associated with enhanced pro-survival signals [188]. This strong expression has also led to considerable interest in SDC1's role in multiple myeloma. Mast cells also express syndecan-4 where it regulates extracellular heparanase uptake [189,190] and eosinophil migration is affected by the absence of syndecan-4 [191]. A summary of the differences in HSPG expression on leucocyte subsets is shown in figure 3.

### 14.3. Heparan sulfate proteoglycans and leucocyte extravasation

The extravasation of leucocytes from the circulation in response to inflammatory stimuli is a tightly regulated process involving multiple steps. These include the initial capture of circulating leucocytes by the endothelium, followed by a rolling phase, eventual arrest and finally extravasation through the endothelium [192]. All of these events involve processes in which the HSPGs have been associated, in particular the syndecans. However, while some studies point to a role for syndecans in this process in various models of inflammation [23,90,122,193], a detailed analysis of the roles of syndecans and glypicans in the leucocyte adhesion cascade have yet to be undertaken. This may in part be due to the unsuccessful clinical trials of HS analogues as anti-inflammatory agents. However, both leucocytes of all types and the endothelium all express at least one HSPG so this avenue of research might well be worth exploring.

### 14.4. Heparan sulfate proteoglycans and angiogenesis

Angiogenesis, the formation of new blood vessels from existing vasculature is distinct from vasculogenesis in which new blood vessels emerge from endothelial stem cell precursors. It is an essential developmental process in animals with a closed vascular system and is also essential for physiological wound healing. Dysregulated angiogenesis in which the process is either upregulated or downregulated is a feature of numerous pathologies and as such has been a target for therapeutic intervention. In both development and disease, angiogenesis is regulated by both pro- and anti-angiogenic growth factors, the prime example being vascular endothelial growth factor A (VEGFA). There is considerable evidence that the majority of these factors can bind to HS and that their activity can be modulated by this interaction. Given this, it is surprising that there are few reports of roles for glypicans in this process with the majority of studies focusing on the syndecans.

**Figure 4.**
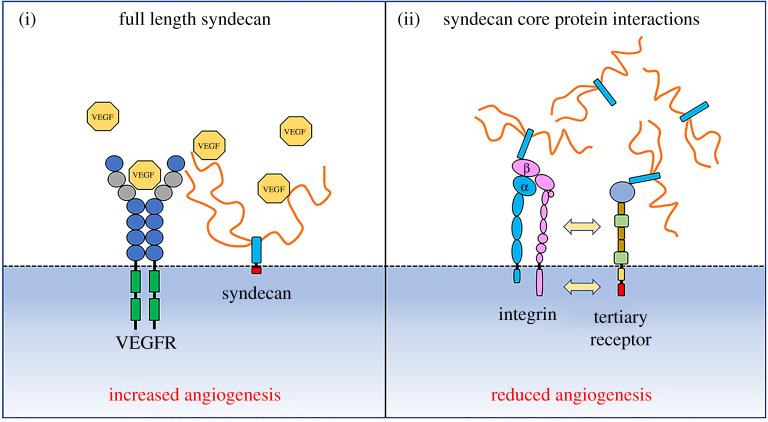
Syndecans have contrasting roles in angiogenesis. On the one hand they have been shown to facilitate the interaction between VEGF isoforms and their receptors (e.g. SDC1, SDC2 and SDC4). However, sequences in the extracellular core proteins of syndecans also have anti-angiogenic properties (SDC1, SDC2 and SDC3). These either act directly on integrins or via the action of tertiary receptors. For references see [39,40,42,122,194–205].

However, the proposed roles for syndecans in angiogenesis present a complex picture with contrasting roles for each family member (figure 4). Despite SDC1 null animals developing a normal vasculature, roles for SDC1 in regulating pro-angiogenic signalling complexes have been identified. Specifically, interactions between integrins, IGFR2, VEGFR2 and VE-cadherin and SDC1 have been characterized and strategies which disrupt these complexes leads to an inhibition of angiogenesis [127,194,195]. Syndecan-2 has been identified as having a role in branching angiogenesis during zebrafish embryonic development and a subtle defect is also observed in endothelial-specific SDC2 knockout mice [40,196]. However, syndecan-2 in its shed form is a potent inhibitor of this process owing to an inhibitory amino acid sequence in its extracellular core protein [197]. Far less is understood regarding syndecan-3, perhaps because its expression has been intimately associated with cells of a neuronal lineage and the musculoskeletal system. However, several studies have identified it as being expressed on endothelium from various vascular beds both *in vivo* and *in vitro* [198,199]. In common with syndecan-1 and 2, regulatory sequences contained within the core protein of syndecan-3 can inhibit EC migration and hence angiogenesis [200]. However, a contrasting role has also been reported whereby thrombin cleaved fragments of the syndecan-3 ectodomain can promote vascular permeability possibly in concert with similar fragments from syndecan-4 [201].

Perhaps the greatest complexity is in understanding the role of syndecan-4 in angiogenesis, particularly as to whether it has a role in the VEGFA/VEGFR2 signalling axis. Despite syndecan-4 null mice developing normally, there is evidence that syndecan-4 has a role in angiogenesis. For example, impaired wound healing in the knockout mouse is in part associated with defects in granulation tissue formation [42]. Knockdown of syndecan-4 in cultured ECs leads to a reduction in VEGFR2 signalling in response to VEGFA, and this is associated with a reduction in angiogenesis-related processes such as EC migration [202]. However, *in vivo* studies comparing a global syndecan-4 knockout mouse with an endothelial cell-specific syndecan-2 knockout mouse suggest that syndecan-4 has no role in VEGFA signalling and it is in fact syndecan-2 which is responsible, particularly during development. This difference in function is related to differences in HS sulfation between the two, with syndecan-2 able to bind VEGFA more effectively due to enhanced levels of 6-O-sulfation [39]. By contrast, during lymphangiogenesis syndecan-4 facilitates the interaction between VEGFC and VEGFR3 during both development and pathological scenarios [203,204]. Angiogenesis is also promoted by FGF-2 and there are a number of studies that indicate a pro-angiogenic role for syndecan-4 in FGF signalling [205–207].

### 14.5. Heparan sulfate proteoglycans in inflammatory disease—rheumatoid arthritis

A number of HSPGs have been identified in various cell types from the inflamed joints of rheumatoid arthritis (RA) patients. Immunohistochemical analysis revealed syndecan-1 expression on infiltrated immune cells in synovia of RA patients and syndecan-2 and -3 were evident on endothelial cells as was glypican-4. Syndecan-2 was also evident on mural cells, and syndecan-3 on macrophages [208]. Despite this, studies investigating the roles of syndecan-1 and -2 in RA are few, and this is also true of glypican-4. Syndecan-2 is associated with bone development but does not appear to have a pathological role in arthritic disease [209]. Syndecan-4 expression was notably absent from the study described above. However, later work revealed syndecan-4 has essential roles in RA pathology. For example, fibroblast-like synoviocytes from RA patients have increased expression of syndecan-4 and showed that ablation of syndecan-4 reduced production of nitric oxide and reactive oxygen species, as well as the production of IL-1β, IL-6 and TNF-α [210]. Syndecan-4 null animals are protected in models of arthritis and disruption of an interaction between syndecan-4 and the protein tyrosine phosphatase receptor PTPR*σ* on fibroblast-like synoviocytes is associated with more severe disease progression in mouse models [211]. During the pathogenesis of RA, significant degradation of cartilage occurs and this is intimately associated with matrix metalloproteinases such as ADAMTS5. In models of osteoarthritis syndecan-4 has been shown to regulate ADAMTS5 activity via interactions with its HS chains and also via transcriptional regulation of MMP3 [43]. The breach of immune tolerance in RA is also a critical step in the early onset of the disease. Syndecan-4 null animals are resistant to collagen-induced arthritis which is T and B cell dependent and this correlated with reduced chemotactic migration in syndecan-4 deficient B cells [212].

In common with syndecan-4, syndecan-3 null mice are protected in models of RA and this is associated with a reduced infiltration of neutrophils into inflamed joints [40]. Administration of a soluble form of syndecan-3 leads to more beneficial outcomes in both antigen-induced and collagen-induced arthritis and again this is associated with inhibition of leucocyte migration [213]. These phenotypes are likely to be linked to the chemokine binding properties of syndecan-3, notably to CCL2, CCL7 and CXCL8 [213,214].

### 14.6. Heparan sulfate proteoglycans in fibrosis

An essential part of normal wound healing is the production of ECM molecules for the restoration of the structural integrity of injured tissues. In circumstances such as chronic inflammation or repeated tissue injury, excessive production of ECM molecules by fibroblasts can occur, leading to scarring and significant interference with an organ's function. ECM production (e.g. collagens and GAGs) is predominantly driven by the pro-fibrotic TGF-β family of growth factors, of which there are three isoforms in mammals. TGF-β is secreted predominantly by macrophages in response to inflammatory stimuli and is produced in complex with LTBP (latent TGF-β binding protein) and LAP (latency-associated peptide). This complex resides in the ECM and in this form TGF-β is not active, it is only when activation either by the action of proteases (e.g. plasmin), physiological changes such as pH or exposure to ROS, inhibition of complex formation by molecules such as thrombospondin-1 or by mechanical disruption through the action of *α*V integrins [215,216]. There are three TGF-β receptors; TGFBR1 and 2 transduce signals upon engagement with TGF-β isoforms, whereas TGFBR3 (betaglycan) acts as a sink sequestering the growth factor via interactions with its GAG chains [217].

Of the HSPGs, syndecan-2 and -4 have the most significant roles in fibrotic disease. Given their roles in focal adhesion formation, which are the sites of matrix deposition, this is not unsurprising. Syndecan-2 has been shown to bind TGF-β and is upregulated in fibrotic tissue and in response to pro-fibrotic stimuli [218,219]. Mice over-expressing syndecan-2 show abrogated radiation-induced lung fibrosis and this is linked to its interaction with the protein tyrosine phosphatase receptor CD148 [220,221]. Syndecan-4 appears to have a protective role in fibrotic disease models since in its absence, outcomes tend to be worse. This has been linked to a number of factors, including syndecan-4 being involved in the abrogation of TGF-β signalling [45] and an interaction with CXCL10 in lung fibrosis [222]. In models of kidney fibrosis, the loss of syndecan-4 resulted in a more severe phenotype, indicating a more protective role associated with reduced activation of the collagen cross-linking enzyme transglutaminase-2 [223].

### 14.7. Cell surface proteoglycans in viral interactions

The polysaccharides of proteoglycans are the most anionic molecules located at cell surfaces by virtue of their sulfate and uronic acid content. A number of different pathogens use these polymers in ionic interactions that locate them to the cell surface, where they may engage with other receptors to gain entry into cells. Additionally, syndecans are known to be effective vehicles for endocytosis [28,29,56]. Over the past 20 years, it has become clear that many different types of virus can interact with HS, and in some cases, these interactions are essential for internalization and pathogenesis that often includes inflammation. A recent in-depth review has summarized the data for over 50 different viruses [224]. However, this review also makes clear that not all have been proven to apply to natural isolates. Examples where this has been demonstrated include Herpes simplex virus (HSV), Dengue virus, Echoviruses 5 and 6 and North American eastern equine encephalitis virus. Many others have shown HSPG dependence based on laboratory strains, or from adaption to cell culture conditions. Some, such as Zika virus and respiratory syncytial virus remain unresolved and require further evaluation.

Much early work focused on HSV, a double-stranded DNA virus and both HSV-1 and -2 attach to the cell surface in a HS-dependent manner. Two viral proteins gB and gC interact with HSPGs that allows translocation on epithelial cells to sites where the main receptors (nectin-1 and -2) and a protein (HVEA) of the TNF family can interact with the viral protein gD [225,226]. Bacsa *et al*. [227] showed that downregulation of both syndecan-1 and sydecan-2 inhibited HSV-1 entry into HeLa cells. Moreover, a form of HS containing 3-O-sulfate residues was found to facilitate gD-mediated internalization [225]. This suggests that a ternary complex of specifically modified HS chains of a syndecan, gD protein and secondary cell surface receptors are responsible for internalization.

It is similarly clear from several studies employing HS deficient cell lines, heparinases or chlorate to suppress sulfation [228], that the four Dengue virus serotypes require interaction with HSPGs at the cell surface. These primary interactions are followed by interactions with known entry proteins, such as DC-SIGN in dendritic cells and the mannose receptor in macrophages [229]. The Dengue virus, an enveloped, single-stranded RNA (ssRNA+) virus is a widespread pathogen, transmitted by *Aedes* mosquitoes, which can lead to haemorrhagic fever and shock syndrome that are potentially lethal.

A third example is that many strains of the human papilloma virus (HPV) bind to cell surface HSPGs. These small non-enveloped dsDNA viruses can infect a range of epithelia with some strains, such as HPV16, being oncogenic and a cause of cervical carcinoma. This strain has therefore been well studied [224]. Syndecan-1 on keratinocytes has been implicated as an initial binding site for these viruses [230], and two lysine residues in the capsid protein L1 have been earmarked as critical [231]. Resulting from HSPG interactions, the HPV capsid undergoes conformational alterations that require cyclophilin B and cleavage of the L2 capsid protein. As a result, an affinity for HSPGs is reduced and secondary receptors, presumably invoking endocytosis become involved. These may include epidermal growth factor receptor (EGFR), integrin *α*6 and tetraspanins [232].

In all these cases, the use of highly anionic competitors, such as heparin and carageenans, can be shown *in vitro* and sometimes *in vivo* to reduce pathogenicity [233]. However, this type of agent has not been translated into successful prevention or treatment, and trials in the case of human immunodeficiency virus (HIV) were not successful [224].

The potential use of competitors including heparin and fucoidans has surfaced again with respect to the current SARS-CoV-2 pandemic. This virus belongs to the coronavirus family, of which there are three major classes based on serological and other criteria [224]. They are enveloped single-strand RNA (ssRNA+) viruses. Previously, the ability of coronavirus to interact with HS was shown after adaptation to culture [224,234]. An example is the human OC43 virus where a mutation in a basic furin cleavage site of the spike protein preserved an HS-binding motif that became obligatory for infectivity [234]. The current SARS-CoV-2 virus has rapidly been shown to possess a heparin-binding site in the spike 1 protein and that sulfated polysaccharides such as heparin and fucoidans inhibited viral entry *in vitro* [235]. HS, with a lower sulfation level than heparin, was, however, ineffective [235]. In further preliminary work, not currently peer-reviewed, both heparin and a commercial low-molecular-weight heparin derivative, Enoxaparin, were effective inhibitors [236]. Other preliminary work suggested that HS octasaccharides could bind the SARS-CoV-2 spike protein, but these were highly sulfated, comprising trisulfated disaccharides [237]. Intriguingly, a further recent report has suggested that cell surface HSPG was essential for infectivity and that spike protein binding to HS and angiotensin-converting enzyme 2 (ACE2) were codependent [238]. In these studies, heparinases or the use of heparin, non-anticoagulant heparin and lung-derived HS could block spike binding and infection [238]. Clearly these studies have a long way to go, but whether syndecans and/or glypicans are essential for SARS-CoV-2 infection is unknown currently. However, two recent reports indicate that neuropilin-1 and -2 can facilitate SARS-CoV-2 entry and infection [239,240]. Neuropilin-1 can possess a HS chain, but whether that is required is not yet established. It will be interesting to ascertain whether there are alterations in HS fine structure or abundance that contribute to the known increased susceptibility of older patients to severe lung disease.

## 15. Concluding remarks

Since the first cDNA cloning of syndecan-1 in 1989 [241], some 4000 publications on these proteoglycans have appeared. They are now implicated in many developmental and disease processes, and for some, such as breast cancer, their presence and distribution can be prognostic [12,13]. In the case of knockout mice, none so far has proved to have serious or lethal repercussions. In lower vertebrates such as zebrafish possessing three syndecans and invertebrates that express one syndecan, developmental defects in mutants are more pronounced. Redundancy across syndecans has only occasionally been clearly demonstrated, but it is apparent that some morphogens and growth factors can functionally bind HS on either syndecans or glypicans. Redundancy between syndecans and glypicans in mammals would appear to be minimal, but a key question that remains is core protein specificity in terms of HS fine structure *in vivo* and its potential impact on protein ligand binding and function.

Of the cell surface proteoglycans, only syndecans appear to work alongside integrins in regulating cell–extracellular matrix interactions, adhesion and cytoskeletal organization. Analysis in *C. elegans* and *Drosophila* points to cell guidance mechanisms and polarity regulation as ancient syndecan functions that can also be demonstrated in mammals. Alongside gene duplications at the invertebrate–vertebrate boundary, additional properties have been acquired in parallel with the acquisition of closed vascular system, extensive skeletal tissues and complex immune systems. Signalling through the V regions of syndecans is still largely unknown, but since invertebrate and mammalian syndecans may regulate stretch-activated calcium channels, this may be a common signalling output impacting the actin cytoskeleton. An important component of the syndecan repertoire is endocytosis, shown to be a key component of lipoprotein uptake in the liver [25] and also in the redistribution of the receptors with which they associate [28]. There is also much to learn regarding syndecan shedding, known to be enhanced in conditions of stress, such as inflammation and diseases where sheddases are upregulated. This may well limit the ability of HS-binding ligands to effect signalling, through competition, but our knowledge is incomplete and may be intrinsic to the progression of a number of diseases. Certainly, the loss of endothelial glycocalyx involves shedding of syndecans into the circulation, and broadly speaking shed syndecan-1 is accepted as a marker of endothelial dysfunction in sepsis and trauma.

It has long been suspected that HS chains can concentrate ligands in the pericellular environment where they may interact with specific receptors. However, this is surely an over-simplification and does not explain why mammals express 10 distinct syndecan and glypican core proteins. Syndecans are transmembrane with linkage to the actin cytoskeleton, a repeated observation. Moreover, this is not simply a mechanism to locate syndecans at the cell surface; syndecan signalling impacts the cytoskeleton, and therefore junctions, migration and pathfinding. Given increasing evidence for roles in development and diseases, including some cancers, musculoskeletal and cardiovascular diseases, their long evolutionary history and widespread tissue expression, it is clear that syndecans continue to deserve scrutiny for several distinct reasons, not least the possibility that they can be diagnostic, prognostic or even targets (e.g. syndecan-1 in myeloma [242]) in specific diseases.

## Data accessibility

This article has no additional data.
